# Combining Fluconazole with Benzo[*a*]phenoxazine Derivatives as a Promising Strategy Against Fluconazole-Resistant *Candida* Species

**DOI:** 10.3390/molecules29215197

**Published:** 2024-11-02

**Authors:** Maria Inês Pacheco, Bárbara Guimarães, Patrícia Pereira-Silva, Augusto Costa-Barbosa, M. Sameiro T. Gonçalves, Maria João Sousa, Paula Sampaio

**Affiliations:** 1 Centre of Molecular and Environmental Biology (CBMA), Department of Biology, University of Minho, Campus of Gualtar, 4710-057 Braga, Portugalmjsousa@bio.uminho.pt (M.J.S.); 2 Centre of Chemistry (CQUM), Department of Chemistry, University of Minho, Campus of Gualtar, 4710-057 Braga, Portugal; 3 Institute of Science and Innovation for Bio Sustainability (IBS), University of Minho, Campus of Gualtar, 4710-057 Braga, Portugal

**Keywords:** *Candida*, benzo[*a*]phenoxazines, fluconazole, synergy

## Abstract

The rise in non-*albicans Candida* species, exhibiting unpredictable antifungal resistance, complicates treatment and contributes to the growing threat of invasive, life-threatening infections. This study evaluates the antifungal activity of four benzo[*a*]phenoxazine derivatives (**C34**, **C35**, **A42**, and **A44**) against 14 *Candida* strains following EUCAST standards. Fluconazole interactions are analysed through fractional inhibitory concentration index (FICI) calculation and response surface analysis based on the Bliss model. Macrophage-like J774A.1 cells are used to assess *Candida* killing in the presence of synergistic compounds. The MIC values against the different strains vary, with **C34** showing the strongest activity, followed by **C35**, while **A42** has the highest MIC values, indicating lower efficacy. However, **A42** demonstrates the best synergy with fluconazole against fluconazole-resistant *Candida* strains. Cytotoxicity assays reveal that the chloropropyl group present in **C35** and **A42** enhances cytocompatibility. Co-culture with macrophages shows significant yeast killing for *C. albicans* and *C. auris* when fluconazole and **A42** are combined, requiring concentrations 4 and 16 times lower than their MIC values, enhancing antifungal activity. Given fluconazole’s fungistatic nature and the emergence of drug-resistant strains, benzo[*a*]phenoxazine derivatives’ ability to enhance fluconazole’s efficacy present a promising strategy to address antifungal resistance in critical pathogens. These findings align with global research priorities, offering new potential avenues for developing more effective antifungal therapies.

## 1. Introduction

*Candida albicans*, the most extensively studied *Candida* species, is a normal coloniser in healthy individuals, typically maintaining commensal interactions with its host [1,2]. However, when its equilibrium with the microbial flora or the host immune system is disrupted, the fungus shifts from a commensal to an opportunistic pathogen [3]. *C. albicans* causes infections that often target mucosal surfaces but can extend its reach beyond mucosal boundaries, leading to systemic or invasive infections that are life-threatening. It is estimated that invasive candidiasis leads to more than 450,000 deaths a year [4]. Several *Candida* species can cause infections in humans, but over 90% of invasive diseases are associated with the same six of them [5]. While *C. albicans* has traditionally been linked to most of these infections, the incidence of non-*albicans Candida* strains has been increasing [6,7]. These strains include *C. krusei*, *C. tropicalis*, *C. parapsilosis*, *C. glabrata* and *C. auris,* as already highlighted in the WHO fungal priority pathogens list [5,8]. The recent rise in non-*albicans Candida* species adds complexity to treatment, given their unpredictable susceptibility to commonly used antifungals [6,7]. This is the case for *C. glabrata*, which has developed resistance to fluconazole and echinocandins during antifungal therapy, or *C. krusei*, which is intrinsically resistant to fluconazole. Moreover, *C. auris* is becoming an increasing problem due to its resistance to the main classes of antifungal agents [9,10].

Benzo[*a*]phenoxazines arise as potential therapeutic approaches due to their established antifungal activity against *S. cerevisiae* [11]. These compounds are Nile Blue analogues with different combinations of substituents in specific positions of the polycyclic core. Positions 2, 5 and 9 are the most common for modifications, although position 10 is also explored [12]. The conjugation of different substituents on these positions, either in terms of the type of substituent group (propyl, chloropropyl, isopenthyl, etc.) or the length of carbon chains leads to benzo[*a*]phenoxazine derivatives with distinct properties. Concerning the antifungal activity, previous works demonstrated that the main players were the substituents on the 5- and 9-positions [11]. In fact, the combination of propyl and dipropyl groups at the amino functions of 5- and 9-positions, respectively—a compound that was named **C34**—favoured the antifungal activity against *S. cerevisiae* compared to other benzo[*a*]phenoxazine derivatives that differed in the length of the aliphatic chains (from 2 to 10 carbon atoms), suggesting an optimal length for the substituents in the mentioned positions [13]. **C34** was also preliminarily tested against different *Candida* spp strains, demonstrating high antifungal activity, even against multi-drug-resistant *C. auris* strains (submitted). Phenoxazine and benzo[*a*]phenoxazine derivatives primarily disrupt DNA functions through intercalation via hydrogen bonding and π-π stacking [14], but functional modifications may also lead to alternative mechanisms of action that may not involve DNA interactions. Some modifications may lead to oxidative stress and cell death, especially in compounds with electron-withdrawing substituents on the phenoxazinone ring, mainly those featuring an iminoquinone function [15]. Additionally, certain derivatives have been shown to directly interact with key cellular molecules, such as by acting as tubulin inhibitors [16]. In silico molecular docking studies have revealed strong binding affinities of several phenoxazine derivatives to microbial intracellular targets, including glucosamine-6-phosphate synthase, AmpC beta-lactamase, and lanosterol-14α-demethylase [17] and may lead to vacuolar membrane permeabilization [18]. Furthermore, *N*-substituted phenoxazines have demonstrated potential as selective allosteric receptor antagonists [11,19]. In our previous studies, we showed that benzo[*a*]phenoxazinium chlorides target vacuolar membranes and, in some cases, the endoplasmic reticulum (ER)’s perinuclear membrane, with docking studies suggesting the ER enzyme oxidosqualene cyclase (OSC, EC 5.4.99.7), a key enzyme involved in the biosynthesis of cholesterol, as a potential target [11]. These examples indicate that the activity of phenoxazine derivatives cannot be defined by a single mechanism, as it is significantly influenced by the specific functionalization of each compound.

Combining two drugs to treat infection offers some potential advantages, including a reduction in the dosage of each compound, thereby decreasing their toxicity and broadening the spectrum of cellular targets, which may bypass resistance [20,21]. Different types of combinations have been presented in the literature with promising results, from combining different clinically used antifungals to using antifungals containing molecules with no previously reported antifungal activity. For example, the combination of isavuconazole with caspofungin against *C. auris* [22],posaconazole with caspofungin against *C. albicans* isolates [23], orcolistin with amphotericin B against different *Candida* species [24], has resulted in synergistic interactions and offering a promising avenue for further exploration.

In a previous work, three compounds featuring different variations in **C34**′s structure were evaluated for their antifungal activity against *S. cerevisiae* **C35**, with the same *N*-di- propyl substituent at the 9-position but with a *N*-chloropropyl group at the 5-position of the heterocycle system [11,25]; **A44** and **A42**, where the free-rotation group at 9-position in **C34** and **C35** was replaced by a rigid structure conferred by a julolidine moiety. Julolidine was chosen, since its cyclic structure is equivalent to the *N*-di-alkylated group of both **C34** and **C35** [26]. Comparative analysis showed that while modifications to **C35** and **A44** did not enhance antifungal activity compared to **C34**, compound **A42**, which combines both alterations, exhibited the highest antifungal efficacy, suggesting that the julolidine nucleus and a chlorine atom at the 14-position positively influence activity [26]. Given the results obtained for *S. cerevisiae*, this work aims to explore the impact of **C34** alteration on the antifungal activity against pathogenic *Candida* spp and explore its potential advantages in combination with fluconazole, particularly in fluconazole-resistant strains, to evaluate the reduction in the dosage of each compound.

## 2. Results

### 2.1. Antifungal Activity of Benzo[a]Phenoxazine Derivatives

As mentioned before, within our research group, five benzo[*a*]phenoxazine derivatives were preliminarily tested against species from the genus *Candida* (submitted). **C34** stood out as the compound that yielded better results, highlighting its potential as an antifungal agent. Following this, we decided to use the same approach to evaluate the activity of a set of compounds with structural variations in **C34**: **C35**, **A44**, and **A42**. **C35** has a chloropropyl group at the 5-position replacing propyl, while **A44** and **A42** feature a rigid structure due to a julolidine moiety, which was chosen for its structural equivalence to **C34**′s di-alkylated group (Table 1). The four compounds were tested against strains of *C. albicans*, *C. glabrata*, *C. parapsilosis*, *C. tropicalis*, *C. krusei*, *C. bracarensis*, *and C. auris.*

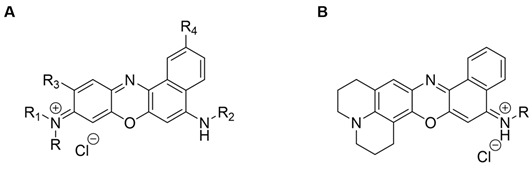



**Table 1:** Benzo[*a*]phenoxazine derivatives used in this study, molecular structures, and MICs against *S. cerevisiae* PYCC 4072. Common positions for synthetic modifications with a free-rotation substituent group (A) and in the presence of a rigid structure, namely julolidine (B), are shown.

Core Structure	Compound	R	R_1_	R_2_	R_3_	R_4_	MIC (µM)	Ref.
**A**	**C34**	(CH_2_)_2_CH_3_	(CH_2_)_2_CH_3_	(CH_2_)_2_CH_3_	H	H	1.56	As 7 [13]
**A**	**C35**	(CH_2_)_2_CH_3_	(CH_2_)_2_CH_3_	(CH_2_)_3_Cl	H	H	6.25	As **C35**/6 [11]
**B**	**A44**	(CH_2_)_2_CH3	-	-	-	-	6.25	As 4c [26]
**B**	**A42**	(CH_2_)_3_Cl	-	-	-	-	0.78	As 4d [26]

After 24 and 48 h of incubation, growth behaviour of the isolates in the presence of the compounds was analysed through calculation of the growth percentage relative to control and determination of the Minimum Inhibitory Concentration (MIC).

**Figure 1:**
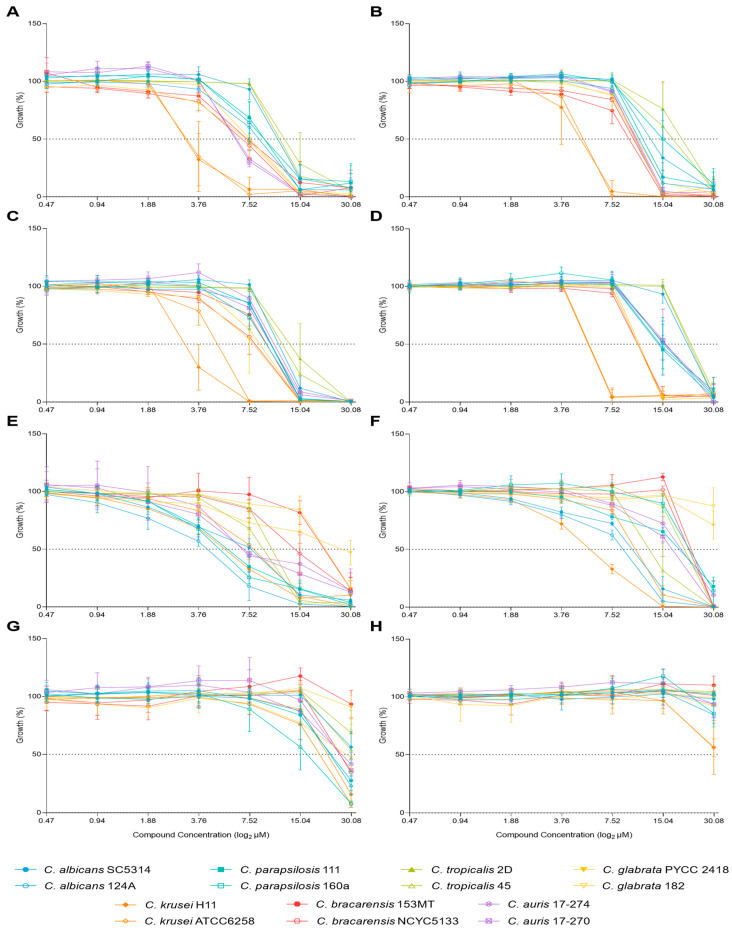
Effect of **C34**, **C35**, **A44**, and **A42** concentrations on the growth of fourteen strains of seven species of *Candida* after 24 h (left) and 48 h (right) of incubation. (**A**,**B**)—**C34**; (**C**,**D**)—**C35**; (**E**,**F**)—**A44**; (**G**,**H**)—**A42**.

The growth percentage was plotted against the compound concentration at both timepoints (Figure 1). Regarding **C34**, the growth profile was similar for all species tested, except for *C. krusei,* which stood out as the most susceptible species following 24 h of incubation, while *C. tropicalis* appeared to be the most resistant species (Figure 1A). These yeasts’ behaviour was maintained after 48 h of incubation (Figure 1B). The trend was similar for **C35**, with *C. krusei* as the most susceptible species, particularly following 48 h of incubation, followed by *C. glabarata* and *C. bracarensis* (Figure 1C,D). Similarly to **C34**, *C. tropicalis* remained the most resistant species, but *C. albicans* SC5314 demonstrated similar resistance (Figure 1D). For **A44**, *Candida* strains displayed a more heterogeneous distribution in terms of susceptibility, even among those from the same species. After 24 h of incubation, *C. albicans* 124A and *C. parapsilosis* 160a were among the most susceptible, while *C. bracarensis* 153 MT and both *C. glabarata* strains stood out as more resistant (Figure 1E). Following 48 h, the trend shows some variations, as *C. krusei* H11 and both *C. albicans* strains revealed themselves to be more susceptible than the others. *C. glabrata* as *C. bracarensis* remained the most resistant to this compound (Figure 1F). As for **A42**, this was the compound to which all species appeared to be, overall, more resistant. *C. parapsilosis* 160a stood out as the most susceptible and *C. bracarensis* 153MT as the most resistant (Figure 1G). This pattern was altered after 48 h, although *C. bracarensis* 153MT remained as the most resistant. The susceptibility profiles of the other strains/species were highly similar, except for *C. krusei*, which was the most susceptible (Figure 1H).

Overall, **C34** and **C35** were more effective than **A42** and **A44**, showing stronger inhibitory effects, particularly against *C. krusei* while *C. tropicalis* and *C. albicans* were the most resistant. **A44** displayed a more heterogeneous response, with *C. albicans* 124A and *C. parapsilosis* 160a being more susceptible and *C. bracarensis* 153 MT and *C. glabarata* strains more resistant. All species appeared resistant to **A42**.

**Figure 2:**
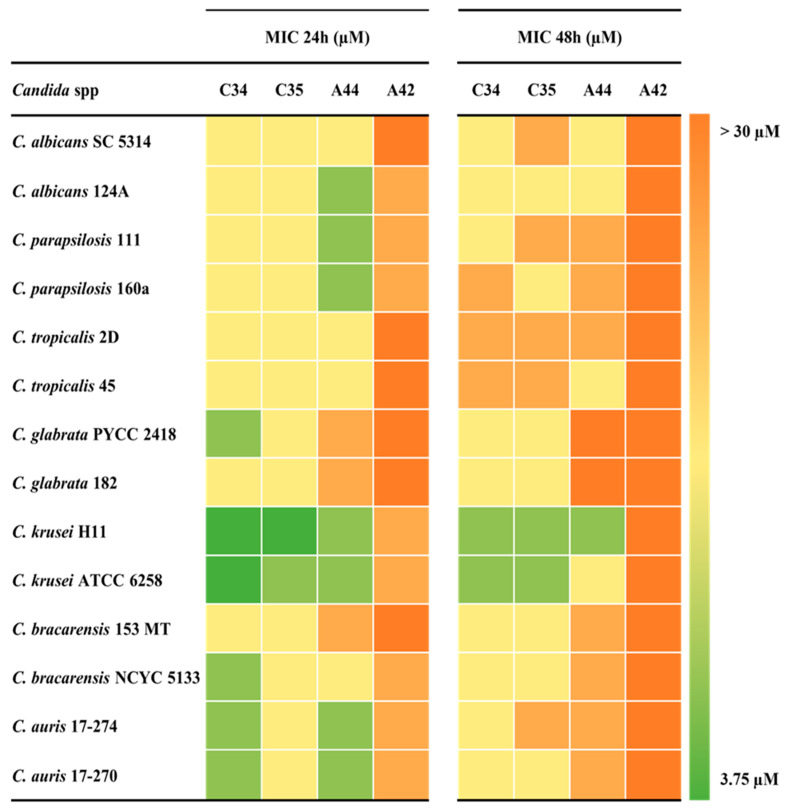
Minimum Inhibitory Concentrations (MICs) of the selected benzo[*a*]phenoxazines against *Candida* species after 24 h (**left**) and 48 h (**right**) of growth, summarised in heat map format. The colour code to the right indicates the range of MIC values obtained.

Figure 2 summarises the MICs values obtained for each strain–compound combination and Table 2 summarizes the associated metrics. The MIC values obtained between replicates varied by no more than one log_2_ dilution.

After 24 h of incubation and considering all *Candida* species tested, the MIC values were very similar for **C34** and **C35**. For these compounds, the values obtained ranged from 3.75 to 15 µM, with MIC geometric means of 10.1 µM and 12.9 µM, respectively. For **A44**, although the MIC values ranged from 7.5 µM to 30 µM, the MIC geometric mean was very close to that of **C35**, with 12.3 µM. On the other hand, **A42** displayed the highest MIC values, indicating that the simultaneous addition of a julolidine moiety and a chloropropyl group, did not contribute to an enhancement of the anti-*Candida* activity (Figure 2 and Table 2).

After 48 h of incubation, minimal deviation was found among **C34** and **C35,** since the MIC values ranged from 7.5 µM to 30 µM in both. Nevertheless, **C34** prevailed as the compound with the lowest MIC geometric mean (MIC GM), followed by **C35**. For both compounds containing the julolidine system (**A44** and **A42**), the MIC values obtained achieved an upper limit of more than 30 µM, with MIC GM close to that value. In this case, it appears that the addition of a rigid structure conferred by a julolidine system decreased the activity of the compounds compared to their free-rotation counterparts (Figure 2 and Table 2).

Overall, our results indicated that while the species exhibited varying responses to the compounds, *C. krusei* was generally the most susceptible, while the most resistant species differed depending on the compound tested. Moreover, **C34** was the most promising compound.

**Figure 3:**
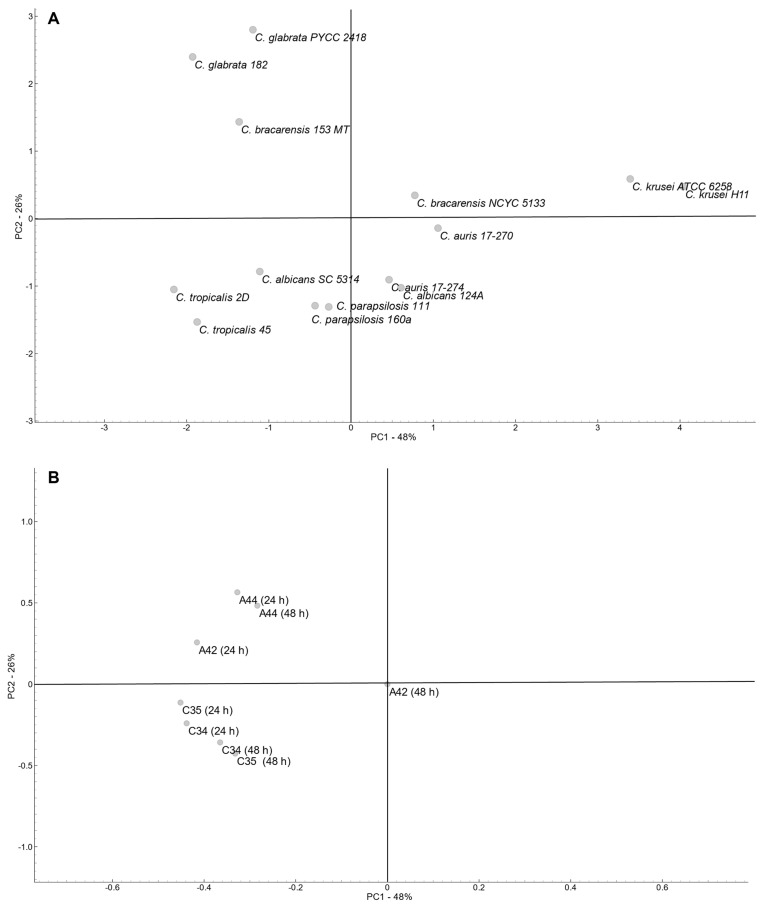
Principal component analysis (PCA) of MICs data from fourteen strains of *Candida* spp. (**A**)—Scores: 14 strains segregation. (**B**)—Loadings: benzo[*a*]phenoxazine derivatives and timepoints tested.

To determine the global profile of variation in all the fourteen *Candida* strains according to the MIC values determined for the four compounds, a principal component analysis (PCA) was performed (Figure 3). Variability was explored considering the first and second PCA components, which explained a total of 74% of the variability (PC1—48%; PC2—26%).

PCA revealed a dispersed arrangement of the strain across the plot. Considering that PC1 explains the higher percentage of variability, a clear separation of both *C. krusei* strains (Group 1) is evident, and *C. auris* strains together with *C. bracarensis* NCYC5133 and *C. albicans* 124A clustered closer (Group 2). Also considering PC1, the remaining strains were positioned close together, but the strong influence of PC2 separated the strains in two groups, one composed of *C. glabrata* strains and *C. bracarensis* 153 MT (Group 3), and the other composed of *C. parapsilosis*, *C. tropicalis* and *C. albicans* SC5314 (Group 4).

Group 1 included the strains with the highest susceptibility to **C34** and **C35** and also susceptible to **A44** at both time points, while group 3 included the strains more resistant to julolidine-based compounds (**A44** and **A42**) and also to **C35** at both time points. The remaining groups include strains with a more heterogeneous profiles, but Group 2 included strains that were susceptible to **C34** and **A44**, particularly at 24 h incubation, while Group 4 included stains that were resistant to **C34** and **C35**, particularly at 48 h of incubation.

In accordance with the strains’ distribution, both free-rotation group compounds (**C34** and **C35**) were clustered separately from the julolidine-based compounds (**A44** and **A42**) at both time points and mainly according to PC2. This suggests a positive correlation among all four compounds, while underscoring the impact of varying substituents on their molecular structures. As expected, since no variability was observed with **A42** after 48 h incubation, this variable was neutral.

### 2.2. In Vitro Antifungal Activity of Fluconazole and Benzo[a]phenoxazine Derivatives

Fluconazole resistance poses a significant challenge in the management of *Candida* infections, requiring alternative or combination therapies [10,27,28,29]. To explore potential solutions and knowing that combining antifungal drugs can combat resistant microorganisms synergistically [20,21], we tested **C34**, **C35**, **A44** and **A42** in combination with fluconazole on fluconazole-resistant strains. To achieve this, we selected four isolates of our collection: *C. albicans* (H65), *C. glabrata* (PYCC2418), *C. krusei* (H11) and *C. auris* (17-274).

**Figure 4:**
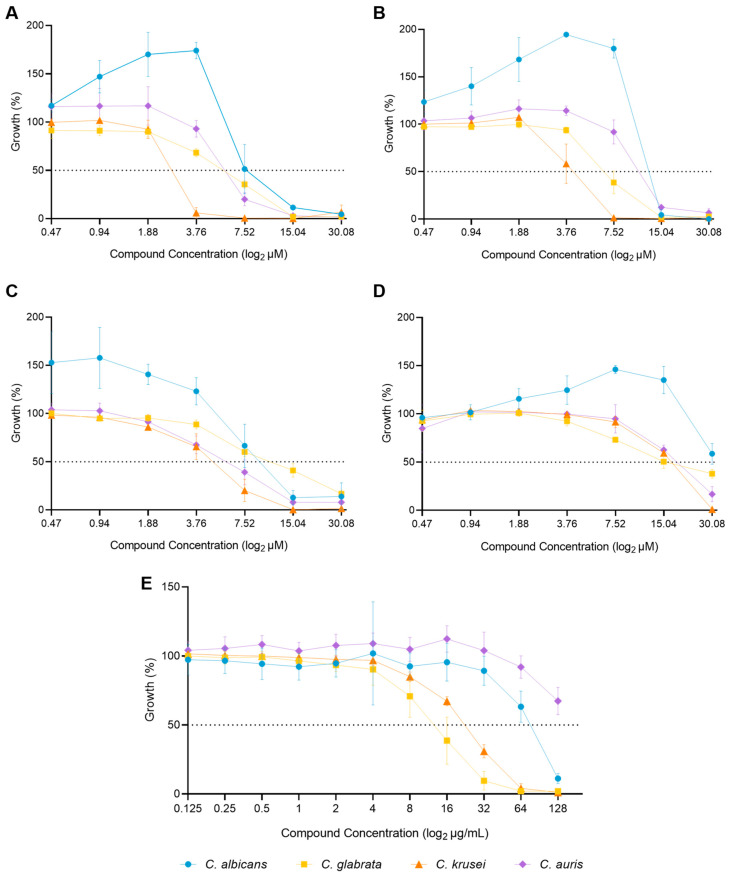
Effect of the concentration of the selected compounds and fluconazole on the growth of four *Candida* species (*C. albicans*, *C. glabrata*, *C. krusei* and *C. auris*). (**A**)—**C34**; (**B**)—**C35**; (**C**)—**A44**; (**D**)—**A42**; (**E**)—Fluconazole.

The selected isolates presented different degrees of susceptibility to the four compounds, with *C. albicans* H65 demonstrating the highest resistance to all four compounds (**C34**, **C35**, **A44**, **A42**) (Figure 4A–D), while resistance to fluconazole varied among the species, with *C. auris* being the most resistant and *C. glabrata* the least resistant (Figure 4E).

The interactions between antimicrobial agents can be analysed by calculating FICI values after checkerboard assays. As mentioned before, interactions are interpreted as synergistic if FICI ≤ 0.5, indifferent if 0.5 < FICI ≤ 4 and antagonistic if FICI > 4. The concentrations of the compounds, as well as the fluconazole used to calculate the FICI, are indicated in each Table (Table 3, Table 4, Table 5 and Table 6) as drug combinations. In this study, we investigated the MIC of fluconazole and each compound both alone and in combination against the four resistant *Candida* species.

The results for **C34** revealed a predominantly indifferent interaction with fluconazole across three out of four species, with FICI values ranging from 0.56 to 1.00. Against *C. krusei*, the MIC of fluconazole decreased from 32 µg/mL to 0.125 µg/mL when combined with **C34**, but the resulting FICI was 1.00, indicating an indifferent interaction. For *C. auris*, the interaction was similar, but in *C. albicans* it was closer to synergism, where the combination reduced the MICs of both drugs, resulting in an FICI of 0.56. On other hand, for *C. glabrata*, the MIC of fluconazole increased from 16 µg/mL to 64 µg/mL when combined with **C34**, with FICI exceeding 4.00, suggesting an antagonistic interaction, indicating that the combination of these drugs is less effective than either drug alone (Table 3).

For the fluconazole–**C35** combination, the results predominantly indicated indifferent interactions, showing FICIs above 0.50. Against *C. glabrata*—the species for which the combination with **C34** resulted in antagonistic interaction—the combination yielded a FICI of 0.56, with the MIC of fluconazole at 16 µg/mL and **C35** at 7.5 µM individually, decreasing to 8 µg/mL and 0.47 µM, respectively, when combined. However, an exception was observed with *C. krusei*, where the combination of both drugs resulted in a FICI of 0.50, indicating a synergistic interaction, with the MICs of both drugs being reduced (Table 4).

The combination of fluconazole with **A44** yielded a markedly different interaction profile, especially compared to its free-rotation counterpart (**C34**). In this case, for *C. albicans*, *C. krusei* and *C. auris,* the interactions were synergistic. Against *C. albicans,* the FICI obtained was 0.31, indicating a significant reduction in MIC values when both drugs are used together. Similarly, *C. krusei* and *C. auris* showed FICIs of 0.38, highlighting strong synergistic effects. For *C. glabrata*, the interaction was indifferent (FICI 0.53) (Table 5).

Remarkably, the combination of fluconazole and **A42** resulted in synergistic interactions across all tested *Candida* species. Against *C albicans*, a substantial reduction in the MIC values for both drugs was observed, with an FICI of 0.26. Similarly, *C. krusei* and *C. auris* displayed FICIs of 0.31, and even *C. glabrata*, which showed indifferent or antagonistic interaction with the other compounds, exhibited a FICI of 0.38 with the combination with **A42** (Table 6).

These results suggest that the combination of fluconazole with julolidine-based compounds, particularly **A42**, is more efficient than their counterparts, which may provide a highly effective option, resulting in synergy against fluconazole-resistant *Candida* strains.

While informative, it is important to note that FICI analysis has some limitations, such as being dependent on an inhibition endpoint—in this case, 50%. Therefore, it only considers data corresponding to the MIC, rather than all the data generated. To gain a more comprehensive understanding of these interactions, we used response surface modelling (RSM) analysis, which allows the calculation and visualisation of the interaction between drugs, plotting the combined effects on a three-dimensional surface. Since this approach considers all data, it is possible to achieve a more nuanced interpretation of the relationships between fluconazole and each compound beyond what is captured by FICI alone. Considering the RSM, the expected effect of a drug combination is based on the individual performance of each drug. This predicted outcome is then compared to the obtained experimental results and if the combined effect is greater than, equal to, or less than predicted, the interaction is classified as synergy, indifference, or antagonism, respectively.

**Figure 5:**
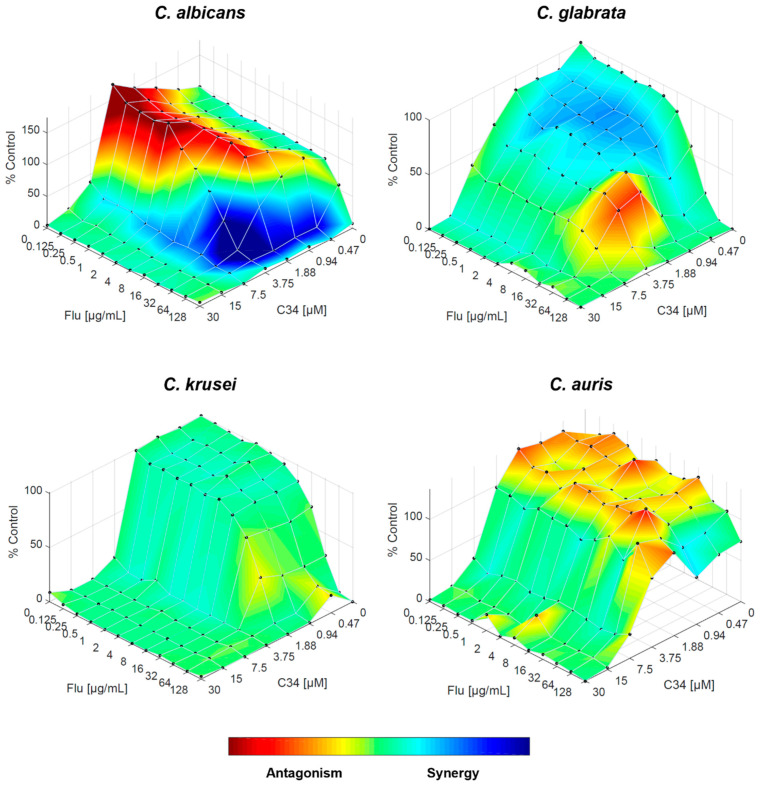
Combination of **C34** and fluconazole against *C. albicans*, *C. glabrata*, *C. krusei* and *C. auris*, analysed by response surface modelling based on the Bliss model. Synergy is mapped on the experimental response surface.

Regarding the combination of fluconazole and **C34**, the FICI indicated an indifferent interaction for *C. albicans*, but the RSM analysis revealed a more complex interaction pattern. While the overall interaction might be indifferent, there are specific concentration ranges in which the drugs interact synergistically or antagonistically, as shown by the areas of blue and red on Figure 5, respectively. For *C. glabrata*, for which FICI showed an antagonistic interaction, the RSM analysis demonstrated that antagonism occurs at specific concentrations, but its effect is not uniform across all concentrations, and there are even regions of weak synergism. For *C. krusei*, both approaches indicated indifference, and for *C. auris*, although the overall interaction is indifferent, there are isolated concentrations where antagonism might occur (Figure 5).

**Figure 6:**
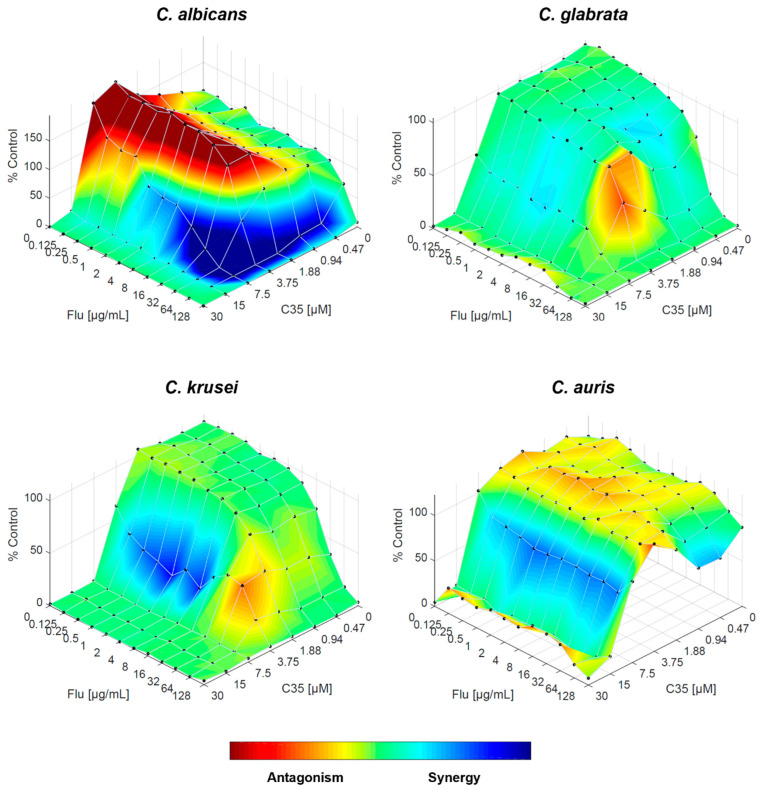
Combination of **C35** and fluconazole against *C. albicans*, *C. glabrata*, *C. krusei* and *C. auris*, analysed by response surface modelling based on the Bliss model. Synergy is mapped on the experimental response surface.

When combining fluconazole with **C35**, the effect on *C. albicans* was highly similar to the combination with **C34**; however, despite the FICI suggesting an indifferent interaction, the actual interaction appears more complex. For *C. glabrata*, the indifferent interaction observed with FICI is established, although there might be minor antagonistic effects. For *C. auris*, the scenario is inverted: while the overall interaction remains indifferent, some drug concentration ranges begin to interact synergistically, and antagonistic effects are less prominent. Finally, the synergistic interaction observed for *C. krusei* is supported by this approach (Figure 6).

**Figure 7:**
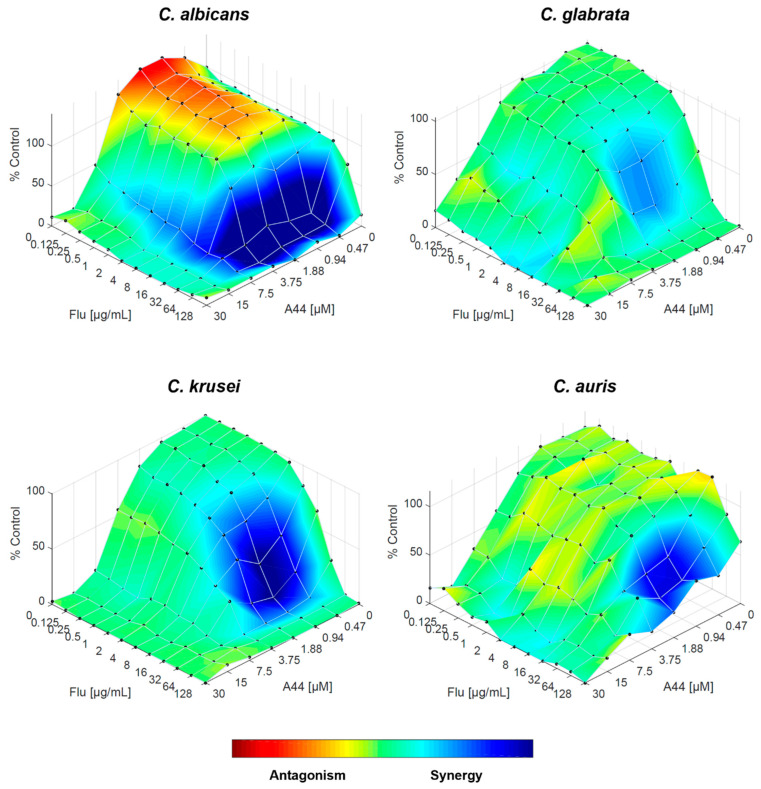
Combination of **A44** and fluconazole against *C. albicans*, *C. glabrata*, *C. krusei* and *C. auris*, analysed by response surface modelling based on the Bliss model. Synergy is mapped on the experimental response surface.

The fluconazole–**A44** combination resulted—according to FICI—in synergistic interaction for all species, except for *C. glabrata*. This was confirmed by RSM analysis, especially for *C. krusei* and *C. auris*, as the area of synergy is more prominent than with other compounds. For *C. albicans*, although there are still specific concentration ranges in which the drugs interact antagonistically, the effect is weaker compared to the effects on **C34** and **C35**, and the overall trend is considered to be synergistic. The interaction of both drugs in *C. glabrata* is mostly indifferent, but the weak synergy, along with the absence of antagonism, holds promise (Figure 7).

**Figure 8:**
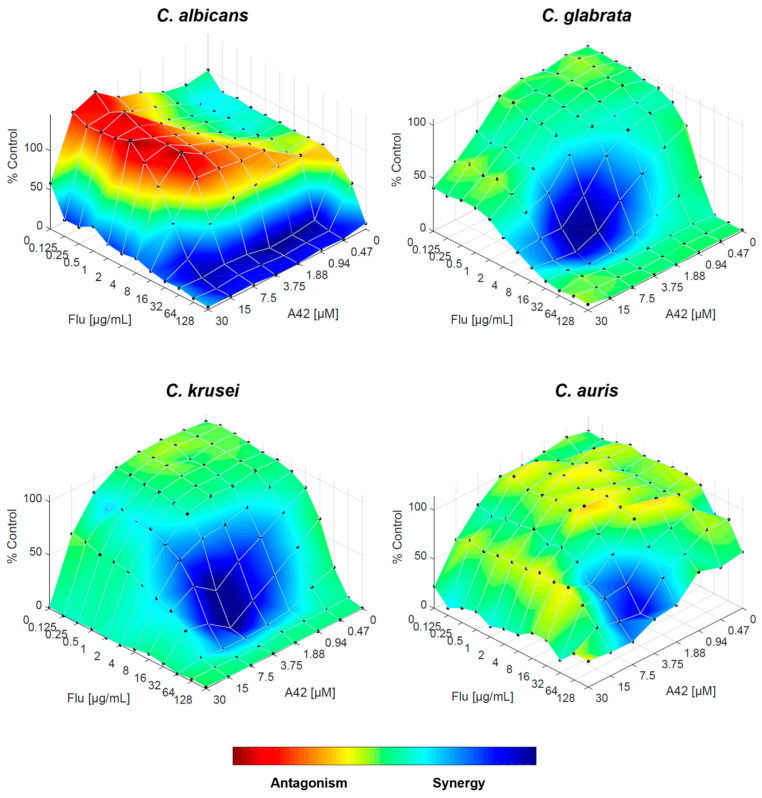
Combination of **A42** and fluconazole against *C. albicans*, *C. glabrata*, *C. krusei* and *C. auris*, analysed by response surface modelling based on the Bliss model. Synergy is mapped on the experimental response surface.

Finally, when fluconazole and **A42** are combined, the RSM analysis reinforces the FICI results, with synergistic interaction across all *Candida* species tested. For all four species, the trend is strongly synergistic, even in *C. glabrata*, confirming that the combination of both drugs works effectively (Figure 8).

While FICI provided a summary measure of interaction, RSM analysis revealed specific concentration-dependent interaction patterns. Therefore, combining these two approaches allows for a better understanding of the complexity of drug interactions and offers a framework for optimising drug combinations for clinical use.

Moreover, the agreement between FICI and RSM analysis results indicates that the compounds containing the julolidine moiety (**A44** and **A42**) lead to a greater synergistic interaction with fluconazole than their free-rotation counterparts (**C34** and **C35**).

### 2.3. Cytotoxicity Assessment

**Figure 9:**
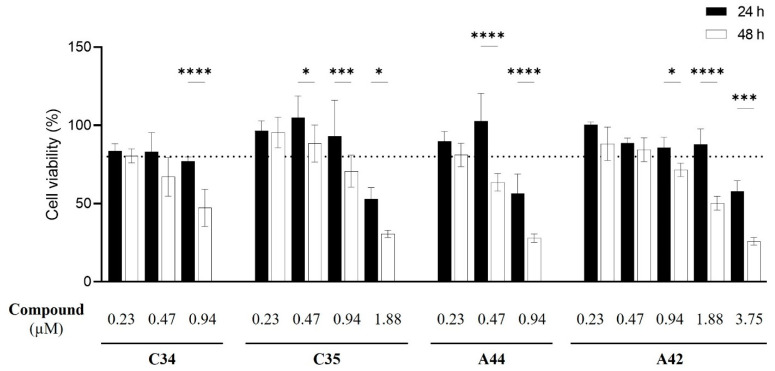
Effect of increasing concentrations of each benzo[*a*]phenoxazine (**C34**, **C35**, **A44** and **A42**) on the viability of J774A.1 cells. Viability was assessed using the MTT assay after 24 h (black bars) and 48 h (white bars) of incubation. The results indicate the mean ± SD of three independent assays. Significant differences between the two time points of incubation, 24 h and 48 h, are represented by * *p* < 0.05, *** *p* < 0.001, **** *p* < 0.0001.

Following the assessment of the in vitro interaction of these compounds with fluconazole, it became necessary to assess their cytotoxicity to determine their potential in vivo use and identify the most promising compound for further development. The MTT assay was used to test increasing concentrations of each compound to determine the concentration at which they become cytotoxic for the macrophage-like cell line J774A.1 (Figure 9).

The results showed that, for **C34** and **A44,** the highest non-cytotoxic concentration following a 24 h incubation period was 0.47 µM of compound, with only 0.23 µM maintaining that profile following 48 h. For **C35**, the highest non-cytotoxic concentration was 0.94 µM at 24 h, after which noticeable cytotoxicity was observed when increasing the concentration of the compound. Remarkably, **A42** was non-toxic at concentrations up to1.88 µM at 24 h and at 48 h, the maximum non-toxic concentration was 0.47 µM. These results suggest the incorporating Cl as a terminal might enhance the cytocompatibility of these compounds with macrophage-like cells. Nevertheless, non-toxic concentrations of all compounds were below the *Candida* MIC values.

Overall, cytotoxicity results highlighted **A42** as the least cytotoxic compound. Given that the data on the antifungal interaction with fluconazole also identified this compound as the most promising against resistant *Candida* species, we used **A42** to proceed with the next steps.

### 2.4. Macrophage Yeast Killing

Integrating all the results, namely FICI and RSM analysis, with cytotoxicity, we proceeded to evaluate whether the combination of **A42** and fluconazole could enhance the antifungal activity of macrophages. Considering the fungal burden in the presence of macrophages without any drug treatment as our baseline, we assessed the fungal survival in the presence of the phagocytic cells incubated with fluconazole and **A42** individually and in combination in each of the previously tested resistant *Candida* species (*C. albicans*, *C. glabrata*, *C. krusei* and *C. auris*). The concentrations of fluconazole used in this assay were selected for each species as the lower concentrations where the drugs might interact synergistically, and we also considered the highest non-toxic **A42** concentration at 24 h of incubation. Thus, for *C. glabrata,* fluconazole was tested at 4 µg/mL (MIC 16 µg/mL), *C. krusei* at 8 µg/mL (MIC 32 µg/mL), *C. albicans* at 32 µg/mL (MIC 128 µg/mL) and *C. auris* at 64 µg/mL (MIC > 128 µg/mL). **A42** was tested at 1.88 µM and the MIC values for all species were ≥30 µM. These concentrations were chosen based on the RSM analysis, and concentrations were selected within the synergistic combination (Figure 5, Figure 6, Figure 7 and Figure 8).

**Figure 10:**
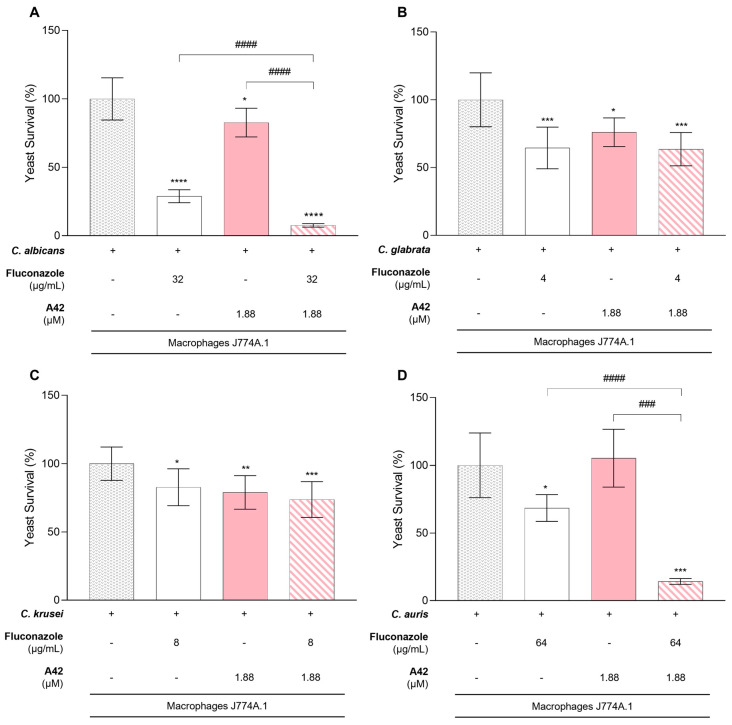
Results of macrophage killing assay using the cellular line J774A.1 and four fluconazole-resistant *Candida* species. (**A**)—*C. albicans*; (**B**)—*C. glabrata*; (**C**)—*C. krusei*; (**D**)—*C. auris*. These graphics compare the yeast survival after incubation with macrophages, macrophages with fluconazole or **A42** (alone) and macrophages with the combination of both drugs to evaluate the synergistic effect. The results indicate the mean ± SD of two independent assays. Significant differences with *Candida* cells incubated with macrophages are represented by * *p* < 0.05, ** *p* < 0.01, *** *p* < 0.001, **** *p* < 0.0001. Significant differences between the single-drug effects and the effects of the multi-drug combinations are represented by ### *p* < 0.001, #### *p* < 0.0001.

For *C. glabrata* and *C. krusei*, although the presence of fluconazole and **A42** alone resulted in a reduction in yeast cell survival, the combination of fluconazole and **A42** did not lead to a notable reduction in yeast survival. This indicated that in *C. glabrata* and *C. krusei,* the combination did not enhance the antifungal activity of macrophages beyond the effect of individual drugs, despite the in vitro indications of synergistic interaction (Figure 10B,C).

Interestingly, *C. albicans* and *C. auris* demonstrated a different trend (Figure 10A,D). For *C. albicans*, the presence of fluconazole alone led to a significant reduction in yeast survival to 26% while with **A42** alone, the yeast survival only reduced to 83%. Notably, for the combination of both drugs, the decrease in fungal burden was significant comparing to control and to single-drug treatment groups, leading to only 8% yeast survival (*p* < 0.0001) (Figure 10A). For *C. auris,* the presence of fluconazole alone reduced the yeast survival but only to 68%; however, contrarily to *C. albicans*, the presence of **A42** alone did not reduce cell survival. Nevertheless, the combination of both drugs led to a significant reduction in yeast survival to only 14% (*p* < 0.0001) (Figure 10D).

The concentrations of drugs used in this assay were significantly lower than their respective MIC values for both fluconazole and **A42** yet, for *C. albicans*, the fluconazole concentration employed still led to significant growth inhibition (71%) (Table 7). For the other species, the growth inhibition observed was less impressive. For **A42**, the non-toxic concentration was approximately 16-fold lower than the MIC values obtained for each species and did not achieve similar levels of inhibition. However, when the two compounds were combined, for *C. albicans* and *C. auris*, growth inhibition was notably enhanced. For *C. albicans*, the combination of compounds resulted in 92% growth inhibition, an improvement of 21% compared to the single treatment with fluconazole. For *C. auris*, the 86% growth inhibition observed with the combination of fluconazole and **A42** was particularly remarkable, representing a 54% improvement over the inhibition achieved with fluconazole alone. *C. glabrata* and *C. krusei* did not exhibit significant improvement compared single-drug treatment.

Taking this into account, the combination of fluconazole and **A42** within the non-toxic concentration limits offers a promising strategy to enhance macrophage-mediated antifungal activity against *C. albicans* and *C. auris*. For *C. glabrata* and *C. krusei*, the lack of enhanced activity requires further investigation.

## 3. Discussion

In this work, we evaluated four benzo[*a*]phenoxazine derivatives against *Candida* species to evaluate their potential as antimicrobials. We evaluated **C34** and three of its derivatives: **C35**, with a chloropropyl group replacing a propyl at the 5-position amino of the heterocycle system, as well as **A44** and **A42**, for which the free-rotation group at 9-position in **C34** and **C35** was replaced by a rigid structure conferred by a julolidine moiety. The overall range of MIC values was very similar among the four compounds, except for **A42**, which exhibited the highest MIC values, followed by **A44**. Compound **C34** showed the best antifungal activity with the lowest MIC geometric mean, closely followed by **C35**. These same compounds had been previously tested against *S. cerevisiae* and **C34** also displayed better activity compared to **C35** and **A44**; however, **A42** emerged as the best compound, indicating that combining the rigid julolidine system in the chloropropyl group enhanced antimicrobial activity [11,26]. In *Candida* species, the results were quite different. Overall, the species tested appeared to be more resistant to the compounds than *S. ceverisiae*. Considering all four compounds, the best antifungal activity was exhibited by **C34**, with **A42** appearing to be the less effective compound, which was unexpected. One of the major challenges in treating fungal infections is the emergence of antifungal resistance among pathogenic fungi. *C. albicans* employs two primary strategies to develop antifungal resistance: (i) Alterations in drug targets, as was the case for fluconazole-resistant strains; the 14-α-sterol demethylase is encoded by the ERG11 gene. (ii) Enhanced PDR efflux pumps such as the overexpression of the CDR1 and CDR2 genes, which encode ABC transporters, as well as the CaMDR1 gene, which is a major facilitator. Since these mechanisms of drug resistance are also present in *S. cerevisiae*, the differences in susceptibility between the species used in this study and between *S. cerevisiae* may be explained by differences in the expression of the drug efflux transporters or differences in the binding affinities to drug targets [30]. Previous studies indicated that the benzo[*a*]phenoxazine **BaP1** in *S. cerevisiae* induces cell death through vacuolar membrane permeabilization and is not a substrate of PDR efflux pumps [18], with similar compounds **C19** and **C20** staining the vacuolar membrane with varying degrees of affinity [11]. Khandelwal et al. proposed a vacuole-based azole resistance strategy involving the Mlt1 transporter, which sequesters azoles to reduce cytoplasmic concentration [31]. In addition, molecular docking studies suggested the enzyme oxidosqualene cyclase, a key enzyme involved in the biosynthesis of cholesterol, as potential target [11]. This enzyme is present both in mammals and yeasts and is important for lanosterol formation in sterol biosynthesis. Although **BaP1**, **C19** and **C20** share a benzo[*a*]phenoxazine core with other derivatives, unique substitutions influence their vacuolar affinity and even their affinity for oxidosqualene cyclase, including the four compounds in this study [20,21,22]. Taking our results into account, we selected four fluconazole-resistant *Candida* to test the combination of this well-known drug with these four compounds. Surprisingly, **A42** emerged as the compound with the best performance when used in combination, leading to synergistic interactions in all tested species. This was equally supported by the results obtained with the calculation of the FICI and response surface analysis based on the Bliss model. Recent studies have focused on the interactions of fluconazole with new compounds against *Candida* species. For example, Kane and co-workers (2024) tested bisphosphonates against different *Candida* species isolates, along with their interaction with fluconazole, from which a promising combination emerged [32]. Similarly, Albuquerque et al. (2024) explored molecular hybrids of aza-bicyclic 2-isoxazoline-acylhydrazone, a similarly heterocyclic compound, as anti-*Candida* agents, revealing one hybrid that—when combined with fluconazole—had synergistic effects on a fluconazole-resistant *Candida* strain [33]. These findings underscore the importance of developing new strategies, either through developing novel compounds or exploring their combinations, to improve antifungal activity and enhance treatment effectiveness, with heterocyclic compounds showing considerable promise.

To consider the applicability of the benzo[*a*]phenoxazine derivatives under study, it was necessary to evaluate their cytotoxicity. The results showed that the presence of the chloropropyl group in **C35** and **A42** appears to be essential to the cytocompatibility of these types of compounds compared with **C34** and **A44**. Moreover, its combination with the julolidine system further improved the cytocompatibility, highlighting compound **A42** as the best one to combine with mammalian cells.

Co-culturing of fungi with macrophages in the presence of promising compounds is a common method for evaluating how the compounds influence fungal vulnerability to host immune defences. In our group, this approach has been used to assess the ability of macrophages to kill *Candida* cells in the presence of selected phytocompounds, either in their free form or while encapsulated [34], with the latter demonstrating superior activity. More recently, Gutierrez-Gongora et al. (2024) [35] used this approach to test mussel extracts in co-cultures of *C. neoformans* and macrophages, proving their efficacy in reducing fungal survival, both on their own and in combination with fluconazole. In the present work, results obtained from the killing of macrophage yeast showed a significant decrease in yeast survival, namely for *C. albicans* and *C. auris*, when fluconazole and **A42** were used in combination compared to their single forms. It is worth noting that the concentrations of drugs responsible for these results were 4- and 16-fold lower, respectively, than the MIC value for the species and remained capable of potentiating the antifungal activity of both compounds. Fluconazole is known for its fungistatic nature, paving the way for the development of resistance among isolates [10,36,37]. Efflux pumps, particularly those mediated by ATP-binding cassette (ABC) transporters (Cdr1p and Cdr2p) and the major facilitator superfamily (MFS) transporter (Mdr1p) are often associated with azole resistance in *C. albicans* [38]. Interestingly, Nile Red, which also belongs to the benzophenoxazine family, has been reported as a fluorescence substrate of these transporters [39], suggesting that these types of compounds could potentially inhibit them, thereby enhancing the activity of fluconazole by increasing its intracellular accumulation [36]. In addition, the in silico molecular docking studies revealed the strong binding affinities of several phenoxazine derivatives to lanosterol-14α-demethylase [17], promoting intracellular accumulation of 14α-methyl sterols. Curiously, it appears that cells with 14α-methyl sterols, which accumulate in fluconazole-treated cells, have an increased sensitivity to the oxygen-dependent microbicidal systems of the host [40]. Thus, for species that overexpress these transporters (fluconazole-resistant strains), the combined action of fluconazole and the compounds that better bind to the lanosterol demethylase would be more susceptible to this combination.

## 4. Materials and Methods

### 4.1. Fungal Strains and Cell Lines

Fourteen isolates of *C. albicans*, *C. parapsilosis*, *C. tropicalis*, *C glabrata*, *C. krusei*, *C. bracarensis* and *C. auris* were used in this work. These strains belong to the collection of the Biology Department of the University of Minho and were obtained from different sources, identified and conserved in 30% glycerol at −80 °C. When needed, yeast cells were routinely grown at 30 °C on YPD agar medium (Yeast extract 1%, Peptone, 1%, Glucose 2% and Agar 2%).

For cell culture assays, the murine macrophage-like cell line J774A.1 was used. The cell line was cultured in Dulbecco’s modified Eagle’s medium (DMEM; Gibco, ThermoFisher Scientific, Waltham, MA, USA) high glucose supplemented with 10% heat-inactivated foetal bovine serum (FBS; Gibco, ThermoFisher Scientific, Waltham, MA, USA), 2 mM glutamine, 1 mM sodium pyruvate, and 25 mM HEPES buffer, in tissue culture flasks (Nunc, ThermoFisher Scientific, Waltham, MA, USA) in a humified atmosphere with 5% (*v*/*v*) CO_2_ at 37 °C (Binder CB150, Tuttlingen, Germany).

### 4.2. Drugs and Medium

The four benzo[*a*]phenoxazine derivatives **C34**, **C35**, **A44** and **A42** were synthesised at the Center of Chemistry of the University of Minho with a purity higher than 95% according to a previously described procedure [11,13,26]. The preparation of precursors started with the *N*-alkylation reaction of 3-aminophenol with 1-bromopropane in ethanol under reflux conditions. The reaction provided two derivatives, 3-(propylamino)phenol and 3-(dipropylamino)phenol. The 3-(dipropylamino)phenol or 2,3,6,7-tetrahydro-1*H*,5*H*-pyrido [3,2,1-*ij*]quinolin-8-ol (9-julolidinol) were reacted in an acidic solution of sodium nitrite under ice-cold conditions to afford the corresponding 5-(dipropylamino)-2-nitrosophenol hydrochloride or 9-nitroso-2,3,6,7-tetrahydro-1*H*,5*H*-pyrido [3,2,1-*ij*]quinolin-8-ol hydrochloride, respectively.

The other intermediates *N*-propylnaphthalen-1-amine and *N*-(3-chloropropyl)naphthalen-1-amine were obtained by reacting naphthalen-1-amine with bromopropane and 1-bromo-3-chloropropane in ethanol reflux. The condensation of 5-(dipropylamino)-2-nitrosophenol hydrochloride with *N*-propylnaphthalen-1-amine and *N*-(3-chloropropyl)naphthalen-1-amine in the presence of hydrochloric acid, refluxed in ethanol, resulted in *N*-propyl-*N*-(5-(propylamino)-9*H*-benzo[*a*]phenoxazin-9-ylidene)propan-1-aminium chloride **C34** and *N*-(5-((3-chloropropyl)amino)-9*H*-benzo[*a*]phenoxazin-9-ylidene)-*N*-propylpropan-1-aminium chloride **C35**. In the same way, reaction of 9-nitroso-2,3,6,7-tetrahydro-1*H*,5*H*-pyrido [3,2,1-*ij*]quinolin-8-ol hydrochloride with *N*-propylnaphthalen-1-amine or *N*-(3-chloropropyl)naphthalen-1-amine resulted in 3-chloro-*N*-(2,3,6,7-tetrahydro-1*H*,5*H*,14*H*-benzo[*a*]quinolizino[1,9-*hi*]phenoxazin-14-ylidene)propan-1-aminium chloride **A44** or *N*-(2,3,6,7-tetrahydro-1*H*,5*H*,14*H*-benzo[*a*]quinolizino [1,9-*hi*]phenoxazin-14-ylidene)propan-1-aminium chloride **A42**.

Stock solutions were prepared in dimethylsulphoxide (DMSO) to a final concentration of 12 mM. Stock solutions of fluconazole were prepared in DMSO at 25.6 mg/mL. For the susceptibility assays, RPMI 1640 medium (with L-glutamine and a pH indicator but without bicarbonate) was supplemented with glucose to a final concentration of 2% and buffered to pH 7.0 with 0.165 M 3-(*N*-morpholino)propanesulfonic acid (MOPS) (Sigma-Aldrich, St. Louis, MO, USA). Serial dilutions of all benzo[*a*]phenoxazine derivatives and fluconazole were performed in supplemented RPMI 1640 medium and added to 96-well plates to obtain the intended final concentrations.

### 4.3. Minimum Inhibitory Concentration Assays

In vitro susceptibility of all isolates to the four benzo[*a*]phenoxazine derivatives was evaluated according to the European Committee on Antimicrobial Susceptibility testing (EUCAST) protocols for yeasts (Eucast Definitive Document E.Def 7.3.2).

Susceptibility to benzo[*a*]phenoxazine derivatives was evaluated by testing concentrations ranging from 0.47 to 30 μM, while for fluconazole, the concentration varied between 0.125 and 128 μg/mL. Following incubation on YPD agar medium for 24 h at 30 °C, isolates were suspended in sterile water adjusted to a cellular density of 1–3 × 10^6^ cell/mL. Working concentrations of 0.5–1.5 × 10^5^ cell/mL were used. Each microplate included both sterility and growth controls. After 24 and 48 h of incubation at 30 °C, absorbance was measured at 530 nm using a microplate reader. The Minimum Inhibitory Concentration (MIC) of each compound was determined as the lowest drug concentration with growth inhibition of ≥50%, in comparison with the growth control.

### 4.4. Checkerboard Assays—Microplate Preparation

Interaction between fluconazole with each of the benzo[*a*]phenozaxine derivatives (**C34**, **C35**, **A44** or **A42**) followed the same guidelines as the Minimum Inhibitory Concentration (MIC) assays, adjusted to a broth dilution checkerboard approach [41,42], and following the drug dilution scheme recommended by EUCAST. For that, 96-well flat-bottom plates were used. Final concentrations ranged from 0.47 to 30 μM for each of the benzo[*a*]phenoxazine derivatives and 0.125 to 128 μg/mL for fluconazole. For microplate preparation, 50 μL of each concentration of **C34**, **C35**, **A44** or **A42** was added into wells 1 to 11 of each column, while 50 μL of each concentration of fluconazole was added to wells A to G of each line. The wells of column 12 and line H contained each compound and fluconazole alone, respectively. Well H12 served as the growth control, containing RPMI with DMSO, but lacking any antifungal drug. The yeast cell suspensions were prepared in sterile water, as previously described, and each well was inoculated with 100 μL of the suspension. For each combination, an uninoculated blank plate containing sterile water instead of cells was included. The microplates were incubated at 30 °C and after 24 h of incubation, the absorbance was read at 530 nm. The percentage of growth for each well was calculated by comparison with the growth in the drug-free wells using a 50% endpoint of growth inhibition both for drugs tested alone and in combination.

#### 4.4.1. Analysis of Results

MICs that exceeded the highest concentration tested (off-scale MICs) were adjusted to the next highest concentration. To assess drug interaction, the fractional inhibitory concentration index (FICI) and the response surface modelling (RSM) were calculated and analysed.

##### Fractional Inhibitory Concentration Index (FICI)

In all wells corresponding to a MIC, the FICI was calculated as follows:

FICI = FIC A + FIC B = (C_A/_MIC _A_) + (C_B_/MIC _B_),

where C_A_ and C_B_ are the concentrations of drugs A and B when used in combination, and MIC_A_ and MIC_B_ are the concentrations of drugs A and B when used alone. For interpretation, it was considered that if FICI ≤ 0.5, the interaction was synergistic; if 0.5 < FICI ≤ 4, the interaction was indifferent; if FICI > 4, the interaction was antagonistic [43].

##### Response Surface Modelling (RSM)

Response surface modelling is an approach that allows the use of all data instead of a pre-defined endpoint and has been used for testing drug interactions in yeasts, namely those belonging to the genus *Candida* [41,42,44]. Briefly, experimental data are analysed by expressing the growth percentages for each well and fitting dose–response curves for each drug using a Hill equation. Combined with the Bliss independence model—which is used to predict drug effects under the assumption of no interaction—this allows for the prediction of a theoretical response. Comparing the observed experimental results with the theoretical predictions enables the evaluation of drug interactions and the identification of synergistic or antagonistic effects. Therefore, the data relating to the combinations of fluconazole and the compounds were processed using the Combenefit software (2.021 version), allowing visualisation of the interaction [45].

### 4.5. Cytotoxicity Assays

In vitro studies were performed using the murine macrophage-like cell line J774A.1, which was routinely cultured as previously described [46]. Macrophages were seeded on 96-well tissue culture plates (Nunc) at 1 × 10^4^ cells/well and allowed to adhere overnight at 37 °C in a 5% CO_2_ atmosphere. Different concentrations of free compounds were incubated with the cells and cell viability was assessed via the 3-[4,5-dimethylthiazol-2-yl]-2,5-diphenyltetrazolium bromide (MTT) assay [47] after 24 and 48 h of incubation. The results were expressed relative to untreated cells (100% cell viability) [48].

### 4.6. Macrophage Yeast Killing Assays

To evaluate the impact of free benzo[*a*]phenoxazine derivatives and fluconazole on macrophage-mediated killing of *Candida* species, a modified protocol was used [49]. Briefly, 1 × 10^4^ macrophages per well were seeded in 96-well plates (Nunc) and incubated overnight at 37 °C with 5% CO_2_. Chosen concentrations of free compounds and fluconazole were added. *Candida* cells (5 × 10^4^ cells/well) were then added and incubated for 24 h at 37 °C with 5% CO_2_. After incubation, macrophages were lysed with 10% saponin solution, and serial dilutions of the lysates were plated on YPD agar. After 24 h of incubation, colony-forming units (CFUs) were enumerated.

### 4.7. Data Analysis

Data were analysed with ANOVA followed by Šídák’s or Dunnett’s T3 multiple comparisons test using GraphPad Prism (9.0 version, GraphPad Software, Boston, MA, USA). Unless otherwise stated, the results shown are from at least three independent experiments with three replicates each, except for in vitro interactions with fluconazole (FICI, RSM, and macrophage yeast killing), for which the results shown are from two independent experiments, with two to three replicates each. Statistical significance was considered at *p*-value < 0.05. Principal component analysis (PCA) was performed using Orange data mining suite software (version 3.37.0, Ljubljana, Slovenia) to assess the strain variability of *Candida* species following the MIC assays. The analysis, visualisation and quantification of drug combinations regarding the interaction between fluconazole and each compound were performed with Combenefit software (2.021 version, Cambridge, UK) [45].

## 5. Conclusions

Our findings demonstrate that combining fluconazole with benzo[*a*]phenoxazine derivatives may be effective against fluconazole-resistant *Candida* species, *C. albicans* and *C. auris,* suggesting that combining these compounds could be a promising strategy for overcoming antifungal resistance. These are the two *Candida* species among the four ‘critical threat’ pathogens ranked on the WHO report [8], and the research on new and effective antifungal agents, along with possible synergies, is aligned with the identified research and development priorities.

## Figures and Tables

**Table 2:** MIC metrics of the selected benzo[*a*]phenoxazines against *Candida* species after 24 h and 48 h of growth.

Metrics	MIC 24 h (µM)				MIC 48 h (µM)			
	C34	C35	A44	A42	C34	C35	A44	A42
Mode	15	15	7.5	30	15	15	30	>30
Range MIC	3.75–15	3.75–15	7.5–30	30–>30	7.5–30	7.5–30	7.5–>30	>30
MIC GM	10.1	12.9	12.3	40.4	15.8	17.4	24.6	60.0

GM—geometric mean. For GM calculations, off-scale MICs were converted to the next highest concentration.

**Table 3:** Interaction of fluconazole with **C34** against *C. albicans*, *C. glabrata*, *C. krusei* and *C. auris*.

Species	MIC of Drugs Alone		MIC of the Drugs in Combination		Lowest FICI for the Combination	Interpretation
	Flu (µg/mL)	C34 (µM)	Flu (µg/mL)	C34 (µM)		
*C. albicans*	128	7.5	64	0.47	0.56	IND
*C. glabrata*	16	7.5	64	1.88	4.25	ANT
*C. krusei*	32	3.75	0.125	3.75	1	IND
*C. auris*	>128	7.5	0.125	7.5	1	IND

IND—indifference; ANT—antagonism.

**Table 4:** Interaction of fluconazole with **C35** against *C. albicans*, *C. glabrata*, *C. krusei* and *C. auris*.

Species	MIC of Drugs Alone		MIC of the Drugs in Combination		Lowest FICI for the Combination	Interpretation
	Flu (µg/mL)	C35 (µM)	Flu (µg/mL)	C35 (µM)		
*C. albicans*	128	15	64	0.47	0.53	IND
*C. glabrata*	16	7.5	8	0.47	0.56	IND
*C. krusei*	32	7.5	0.125	3.75	0.50	SYN
*C. auris*	>128	15	0.125	15	1	IND

IND—indifference; SYN—synergy.

**Table 5:** Interaction of fluconazole with **A44** against *C. albicans*, *C. glabrata*, *C. krusei* and *C. auris*.

Species	MIC of Drugs Alone		MIC of the Drugs in Combination		Lowest FICI for the Combination	Interpretation
	Flu (µg/mL)	A44 (µM)	Flu (µg/mL)	A44 (µM)		
*C. albicans*	128	7.5	32	0.47	0.31	SYN
*C. glabrata*	16	15	8	0.47	0.53	IND
*C. krusei*	32	7.5	8	0.94	0.38	SYN
*C. auris*	>128	15	64	1.88	0.38	SYN

IND—indifference; SYN—synergy.

**Table 6:** Interaction of fluconazole with **A42** against *C. albicans*, *C. glabrata*, *C. krusei* and *C. auris*.

Species	MIC of Drugs Alone		MIC of the Drugs in Combination		Lowest FICI for the Combination	Interpretation
	Flu (µg/mL)	A42 (µM)	Flu (µg/mL)	A42 (µM)		
*C. albicans*	128	>30	32	0.47	0.26	SYN
*C. glabrata*	16	30	4	3.75	0.38	SYN
*C. krusei*	32	30	8	1.88	0.31	SYN
*C. auris*	>128	30	64	3.75	0.31	SYN

SYN—synergy.

**Table 7:** Summary of the antifungal activity of fluconazole, **A42** and their combination in the presence of macrophages against four fluconazole-resistant *Candida* species.

Species	Fluconazole (µg/mL)		A42 (µM)		Growth Inhibition (%)			Combination Improvement over Fluconazole (%)
	MIC	Tested	MIC	Tested	Flu	A42	Combination	
*C. albicans*	128	32	>30	1.88	71%	17%	92%	21%
*C. glabrata*	16	4	30	1.88	36%	24%	36%	0%
*C. krusei*	32	8	30	1.88	17%	21%	26%	9%
*C. auris*	>128	64	30	1.88	32%	0%	86%	54%

## Data Availability

All original data generated during the study are included in the article in the form of figures and tables. For any further inquiries, please contact the corresponding author.
